# LIN28 upregulation in primary human T cells impaired CAR T antitumoral activity

**DOI:** 10.3389/fimmu.2024.1462796

**Published:** 2024-10-16

**Authors:** Patricia Garcia-Rodriguez, Laura Hidalgo, Miguel Angel Rodriguez-Milla, Beatriz Somovilla-Crespo, Javier Garcia-Castro

**Affiliations:** ^1^ Cellular Biotechnology Unit, Instituto de Salud Carlos III, Madrid, Spain; ^2^ Universidad Nacional de Educación a (UNED), Madrid, Spain; ^3^ Instituto de Investigación de Enfermedades Raras (IIER) & Departamento de Desarrollo de Medicamentos de Terapias Avanzadas (DDMTA), Instituto de Salud Carlos III, Madrid, Spain

**Keywords:** LIN28, *let-7*, CAR T, immunotherapy, osteosarcoma

## Abstract

LIN28, a highly conserved RNA-binding protein that acts as a posttranscriptional modulator, plays a vital role in the regulation of T-cell development, reprogramming, and immune activity in infectious diseases and T-cell-based immunotherapies. LIN28 inhibit the expression of *let-7* miRNAs, the most prevalent family of miRNAs in lymphocytes. Recently it has been suggested that *let-7* enhances murine anti-tumor immune responses. Here, we investigated the impact of LIN28 upregulation on human T cell functions, focusing on its influence on CAR T cell therapy. LIN28 lentiviral transduction of human T cells led to a stable expression of LIN28 that significantly downregulated the *let-7* miRNA family without affecting cell viability or expansion potential. LIN28 overexpression maintained human T cell phenotype markers and functionality but impaired the antitumoral cytotoxicity of NKG2D-CAR T cells both *in vitro* and *in vivo*. These findings highlight the intricate relationship between LIN28/*let-7* axis and human T cell functionality, including in CAR T cell therapy.

## 1 Introduction

LIN28, an RNA-binding protein, is crucial in various cellular processes, such as cell self-renewal, differentiation, metabolism, tissue repair, and reprogramming (1). Initially identified in *C. elegans*, LIN28 is well-conserved among species underscoring its fundamental role (2). Its expression is dynamic, and tightly regulated during undifferentiated cell stages such as embryonic stem cells (3). For that reason, it has been used as a substitute for Yamanaka factors to induce pluripotent stem cells (4). On the other hand, LIN28 is increasingly being acknowledged for its role in the immune regulation of fetal lymphopoiesis.

LIN28 can regulate gene expression by directly interacting with mRNA sequences or blocking miRNA biogenesis, such as the lethal-7 (*let-7*) miRNA family (5). *Let-7* miRNAs have been implicated in regulating the balance between effector T-cells and regulatory T-cell populations (6–8). In human fetal thymus, Lin28b stimulates invariant gd T cells (9). Moreover, studies demonstrate its capacity to reshape hematopoietic progenitors towards fetal-like lymphopoiesis, facilitating the development of different cell subsets, such as gd T lymphocytes and NK cells (10). In adults, murine T cells downregulate *let-7* expression upon antigen stimulation, which promotes proliferation and differentiation of activated T cells into cytotoxic T lymphocytes (CTLs) (11). *Let-7* suppression demonstrates enhanced cytotoxicity of murine CTLs *in vitro*, yet it fails to control tumor growth *in vivo* (11, 12). However, the exact mechanisms by which LIN28 and *let-7* miRNAs act on human T cells are still unknown, including their immunomodulatory properties for potential therapeutic use.

T-cell-based immunotherapy has become a powerful tool to treat infections and cancer. Among these approaches, chimeric antigen receptor (CAR) T-cell therapy has expanded options for oncologic patients (13). Unlike traditional CAR T cells that target a single antigen, NKG2D-CAR T cells are designed to recognize multiple ligands expressed on the surface of a variety of tumor cells (NKG2DL). This broad targeting capability has shown promising efficacy in preclinical models of osteosarcoma (14). Considering this preclinical data, we are evaluating NKG2D-CAR T cell therapy in a phase I clinical trial with sarcoma patients (NCT06087341). Currently, more than 20 clinical trials using NKG2D-CAR as tumor therapy (clinicaltrials.gov).

In the present study, we investigated the impact of LIN28 upregulation on human NKG2D-CAR T-cell functionality, focusing on T-cell viability, expansion, and cytotoxicity. Our results demonstrated efficient overexpression of LIN28 into T cells using lentiviral vectors. Highlighting its regulatory role, LIN28 upregulation significantly downregulates the *let-7* miRNA family in human T cells. Intriguingly, LIN28 upregulation impairs NKG2D-CAR T cell cytotoxicity both *in vitro* and *in vivo*, suggesting a critical role in modulating CAR T cell function and anti-tumor responses. These findings provide crucial insights into LIN28’s impact on human T cell functionality, particularly in the context of CAR T cell therapy.

## 2 Methods

### 2.1 Tumoral cell lines

The human lymphoma cell line K562 and human osteosarcoma (OS) cell lines 143B and Mnng_hOS were cultivated in Dulbecco’s Modified Eagle Medium (DMEM) supplemented with 10% fetal bovine serum (FBS), 100 IU/ml of penicillin/streptomycin, and 2 mM of glutamine. Cell lines were regularly tested for Mycoplasma detection (Lonza). Cells were authenticated by STR analysis (IIBM, Madrid).

### 2.2 Human T cells isolation

Human Leukocyte Reduction System cones were obtained from donors by the Biobank Hospital Universitario Puerta de Hierro Majadahonda (HUPHM)/Instituto de Investigación Sanitaria Puerta de Hierro-Segovia de Arana (IDIPHISA). They were processed under standard operating procedures with the appropriate approval of the Ethics and Scientific Committees. Human peripheral blood mononuclear cells (PBMCs) and Pan T cells were obtained as described previously (15).

### 2.3 Lentiviral vectors, human T cell transduction and *ex vivo* expansion

Lentiviral vectors were produced from the co-transfection HEK293T cells with plasmids encoding pLenti-SFFV-LIN28-PGK-GFP (Abm), PL-SIN-EF1α-EGFP (Addgene) and NKG2D-CAR construct (**Supplementary Figure 2A**) (14), the packaging plasmid (PsPAX2), and the envelope plasmid (VSV-G). The culture supernatants were collected 48 hours post-transfection, filtered through a 0.45 µm filter (Millipore), and subjected to ultracentrifugation at 23,000 rpm for 2 hours at 4°C.

Purified T cells were activated overnight using Dynabeads Human T-Activator CD3/CD28 (Gibco) at a 1:2 bead-to-T cell ratio in X-VIVO 15 media (Lonza) supplemented with 250 U/ml of IL-2 (Miltenyi Biotech). The following day activated T cells were transduced with lentiviral vectors at a multiple of infection (MOI) of 2. Lentiviral vectors were added at the same time in double-transductions.

T cells were transduced with pLenti-SFFV-LIN28-PGK-GFP to obtain Lin28T cells. T cells were maintained at a concentration of 0.5-1x10^6^ in X-VIVO 15 media supplemented with 250 U/ml of IL-2, and the media was refreshed every 2-3 days. From day 5 post-transduction, beads were removed and different combinations of IL2, IL21 and IL15 (PeproTech) were used at 40 U/ml, 100 and 100 ng/ml respectively. Fold change expansion of Lin28T was calculated as the absolute number of T cells in a timepoint divided by the absolute number of T cells at the start of the expansion culture. For CAR studies, T cells were double-transduced with NKG2D-CAR and GFP construct in NKG2D-CAR T cells; and NKG2D-CAR and pLenti-SFFV-LIN28-PGK-GFP (Abm) in NKG2D-CAR Lin28T and maintained as well as T cells. After 5 days, CAR T cells were expanded with IL21 and IL15 at 100 ng/ml concentration. Functional experiments were conducted from day 10 after transduction.

### 2.4 RT-qPCR and Stem-loop RT-qPCR

Total RNAs were extracted from human T cells using RNeasy Mini kit (Qiagen). The stem-loop RT was done as previously described (16). Briefly, cDNA was generated with RETROscript Reverse Transcription Kit (Invitrogen) combined with 1ng of stem-loop RT primers and without them for conventional RT. qPCR was performed using SYBR Select Master Mix (Thermofisher) in QuantStudio (Thermofisher). The sequences of primers used in this study were listed in **Supplementary Table 1**. Expression level of GAPDH was used as an internal control and U6 for miRNA.

### 2.5 Western blot

Total protein was extracted from UTD, Lin28T, NKG2D-CAR T, and NKG2D-CAR Lin28T cells using CelLytic buffer (Merck Sigma) according to manufacturer’s instructions. The proteins were then separated using SDS-PAGE, transferred to PVDF membranes and blocked with 5% milk in TBS. LIN28a rabbit polyclonal (1:500 dilution; Proteintech) and β-actin mouse monoclonal (clone AC-15; 1:100,000 dilution; Merck Sigma) were used as primary antibodies. Secondary antibodies were polyclonal goat anti-rabbit and anti-mouse immunoglobulins conjugated to horseradish peroxidase (HRP; Agilent’s Dako). Chemiluminescent signal was detected using Immobilon Western Chemiluminescent HRP Substrate (Merck Millipore).

### 2.6 Flow cytometry analysis

T cells were analyzed on MACSQuant Analyzer 10 flow cytometer (Miltenyi). Anti–human fluorescence-conjugated antibodies were listed in **Supplementary Table 2**. Viability was determined using 7-amino-actinomycin D staining (Biolegend).

### 2.7 *In vitro* T cell cytotoxicity assay

The cytotoxicity of T cells against K562, 143B and Mnng_hOS were evaluated at the specified effector-to-target (E: T) ratio by performing a 4-hour CyQUANT LDH Cytotoxicity Assay Kit (ThermoFisher) in p96 plate. As well, cytotoxicity of NKG2D-CAR T cells was measured at different ratios (8:1, 4:1, 2:1 and 1:1) against 143B by the previously mentioned test and 8:1 ratio against Mnng_hOS. Cytotoxicity percentages were calculated by (treated LDH activity- spontaneous LDH activity)/(max LDH activity - spontaneous LDH activity) × 100.

### 2.8 *In vivo* studies

OS xenograft models were generated by injecting 1x10^6^ 143B cells subcutaneously in the right flank of NOD.Cg-Prkdcscid Il2rgtm1WjI/SzJ (NSG) mice (Jackson) of 8-12 weeks old. The experimental process and animal welfare were performed at the Instituto de Salud Carlos III in Madrid, Spain. All animal experiments were approved by Institutional Review Board of the ISCIII and Consejería de Medio Ambiente of Comunidad de Madrid (PROEX 133.7/21).

On day 9, the mice were inoculated intravenously with *ex vivo* expanded 3x10^6^ T cells or PBS per mouse as a single dose of treatment. Tumor length (L), width (W), and height (H) were measured with a caliper periodically and tumor volume was calculated as (L × W × H)/2. Tumor growth was calculated by dividing the tumor volume by the initial tumor volume. The endpoint was established when the tumor volume was >1000 mm^3^.

### 2.9 Statistics

Data from the *in vitro* studies were analyzed using a two-way ANOVA comparison test followed by a *post-hoc* test. For *in vivo* studies, a one-way ANOVA with Tukey’s comparison tests was used. Statistical analyses were performed using GraphPad Prism Software V.8.

## 3 Results

### 3.1 LIN28 upregulation preserved the viability and expansion of human T cells *in vitro*

**Figure 1:**
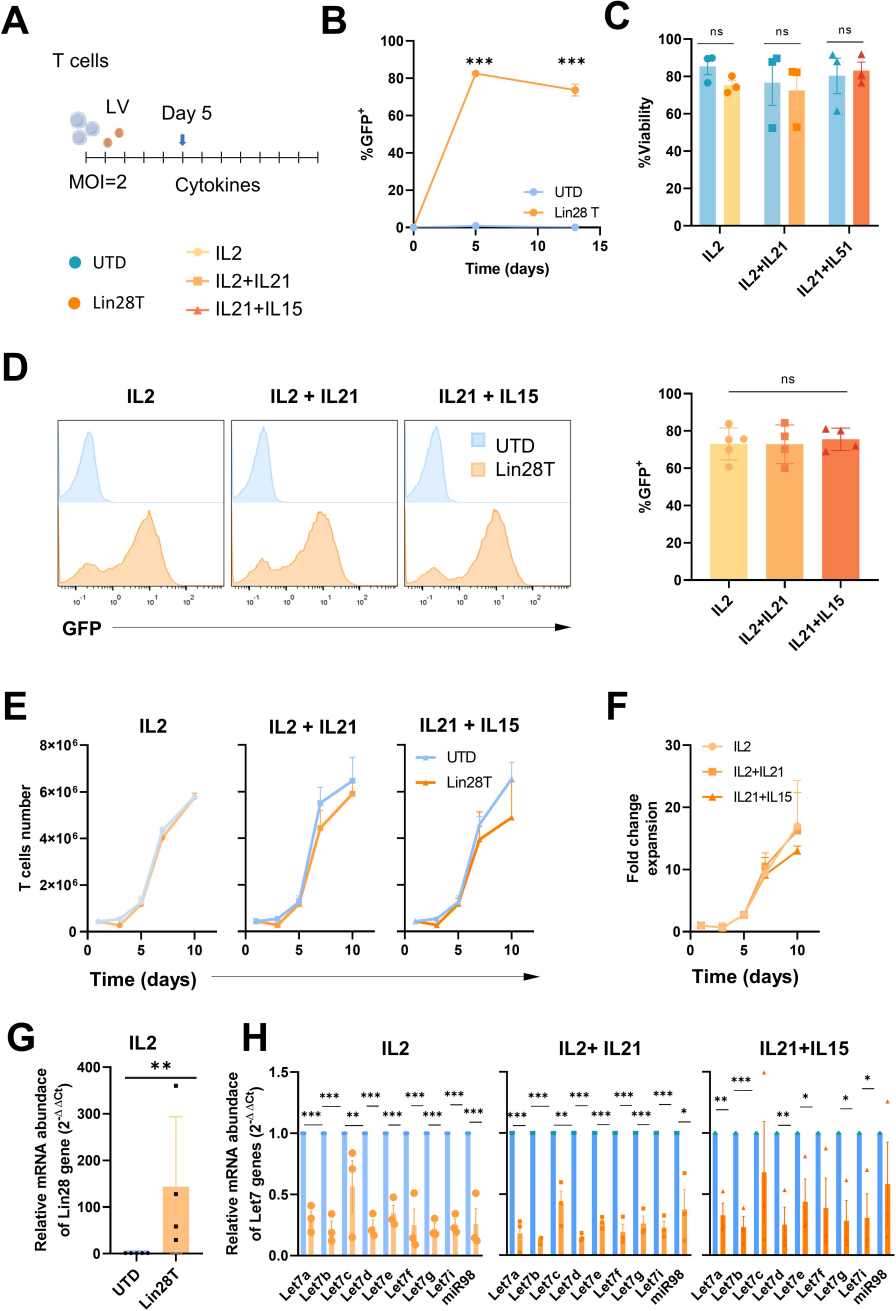
Upregulation of LIN28 down-regulates let-7 miRNAs in human T cells while maintaining their expansion and viability. **(A)** Schematic representation of the activation and transduction regimen for the *ex vivo* expansion of human T cells. **(B)** Lin28 transduction-reporter (GFP) expression was analyzed on days 5 and 12 post-transduction by flow cytometry. **(C)** The viability of Lin28T cells and untransduced cells after cytokines stimulation was analyzed by flow cytometry at day 12. **(D)** Histograms and percentage of GFP expression in transduced T cells after cytokine stimulation at day 12. **(E)** Kinetics of viable T cells overtime. **(F)** Fold change expansion of Lin28T overtime. **(G)** Relative mRNA abundance of the Lin28 gene was quantified by RT-qPCR in T cells expanded with IL2 and at day 12 post-transduction. **(H)** Relative miRNA abundance of the let-7 family was quantified by steep-loop RT-qPCR after different cytokines stimulations. The graphs represent the mean ± SD of three independent experiments (minimum n=3, healthy donors). The (***) indicates p < 0.001, (**) p < 0.01 and (*) p < 0.05, (ns) not significant compared to UTD by t-test analysis. LV: lentiviral vector; UTD: untraduced T cells (blue); Lin28T: LIN28 transduced T cells (orange).

T lymphocytes from healthy human donors were activated and then transduced with a lentiviral vector expressing LIN28 and GFP as reporter gene. After 5 days of lentiviral transduction with LIN28 at MOI 2, we expanded T cells with three different cytokines combinations: IL2, IL2+IL21, and IL21+IL15 (**Figure 1A**). We tested different cytokine stimulations due to their recognized significance in influencing the activation, expansion, and quality of human T cells (17). The efficacy of transduction was first tested by flow cytometry with the IL2 condition at different time points to check whether transduction was stable over time. Our results showed a significant efficient transduction rate. LIN28 upregulation was significant and remained stable over time (**Figure 1B**). On the other hand, neither lentiviral transduction nor expansion with different cytokines combinations affected the viability of T cells (**Figure 1C**). As well, stimulation with different groups of cytokines did not alter LIN28 expression (**Figure 1D**). We counted LIN28 transduced (Lin28T) and untransduced (UTD) cells over time to determine if either cytokines or transduction changed the expansion kinetics of T lymphocytes. Results showed that Lin28T cell expansion *in vitro* followed the expansion of T cells (**Figure 1E**). Overall, similar kinetics of human T cell expansion were found between the different cytokine stimulations (**Figure 1F**).

### 3.2 Upregulation of LIN28 induced downregulation of *let-7* miRNA family in human T cells *ex vivo*

In addition, LIN28 gene expression in transduced human T cells was analyzed by RT-qPCR. A significant increase in the relative abundance of LIN28 mRNA in transduced cells was obtained (**Figure 1G** and **Supplementary Figure 2G**), also in CAR T cells (**Supplementary Figures 2F, G**). To observe whether LIN28 has an impact on miRNAs, we tested the relative expression of the *let-7* miRNA family. We used stem-loop RT-qPCR, a common molecular technique to identify and quantify individual small RNAs. Results showed that the *let-7* miRNA family was significantly downregulated in Lin28T cells after cytokine stimulations (**Figure 1H**) and NKG2D-CAR Lin28T **Supplementary Figure 2H**).

### 3.3 Human T cell phenotype and cytotoxicity were maintained *in vitro* following LIN28 upregulation

**Figure 2:**
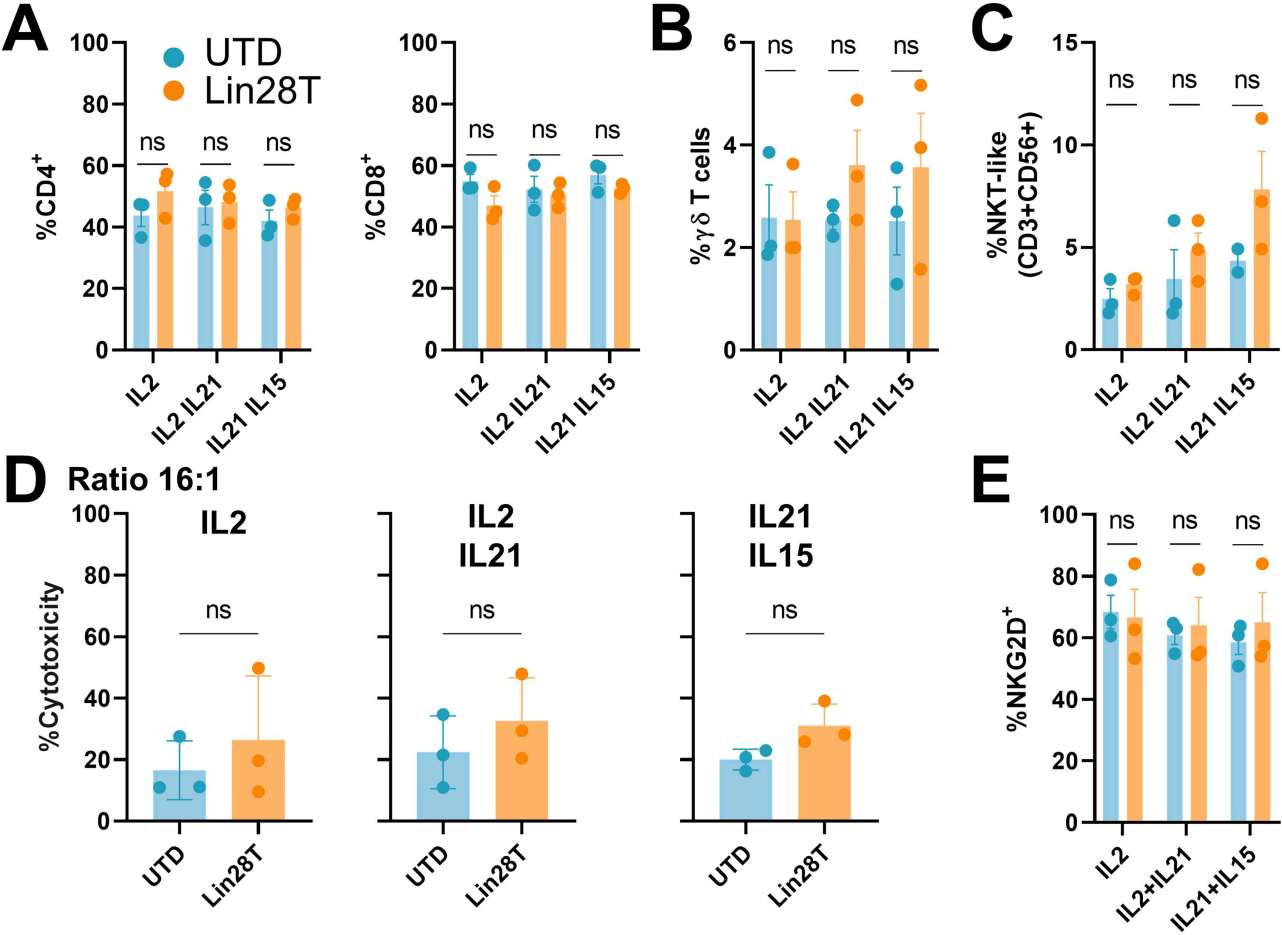
LIN28 upregulated T cells preserve T cells phenotype and cytotoxicity *in vitro*. Lin28T cells phenotype and cytotoxicity were analyzed 12 days after transduction and expansion. **(A)** Percentage of CD4+ and CD8+ T cells, **(B)** gd T cells **(C)** NKT-like cells (CD3+ CD56+). **(D)** Percentage of cytotoxicity measured by LDH assay on K562 cells exposure to T cells in 16:1 effector: target ratio for 4h. **(E)** Percentage of NKG2D+ cells. All data are shown as the mean ± SD. (ns) not significant. All results are representative of at least 3 independent experiments from different healthy donors. UTD: untraduced T cells (blue); Lin28T: transduced T cells (orange).

After activation, lentiviral transduction with LIN28 construct, and expansion with different groups of cytokines, the phenotype of T cells was analyzed by flow cytometry. Results displayed comparable percentages of CD4+ and CD8+ T cells in Lin28T and UTD cell populations (**Figure 2A**). Considering that LIN28 is related to the regulation of T cell development early in life, we assessed whether LIN28 influences unconventional T cell subsets. These immune subpopulations, such as gd T cells and NKT-like cells, revealed no significant differences after LIN28 upregulation neither cytokine stimulations (**Figures 2B, C**). These results maintain that overexpression of LIN28 in human T cells does not affect their phenotype (**Supplementary Figure 1A**).

**Figure 3:**
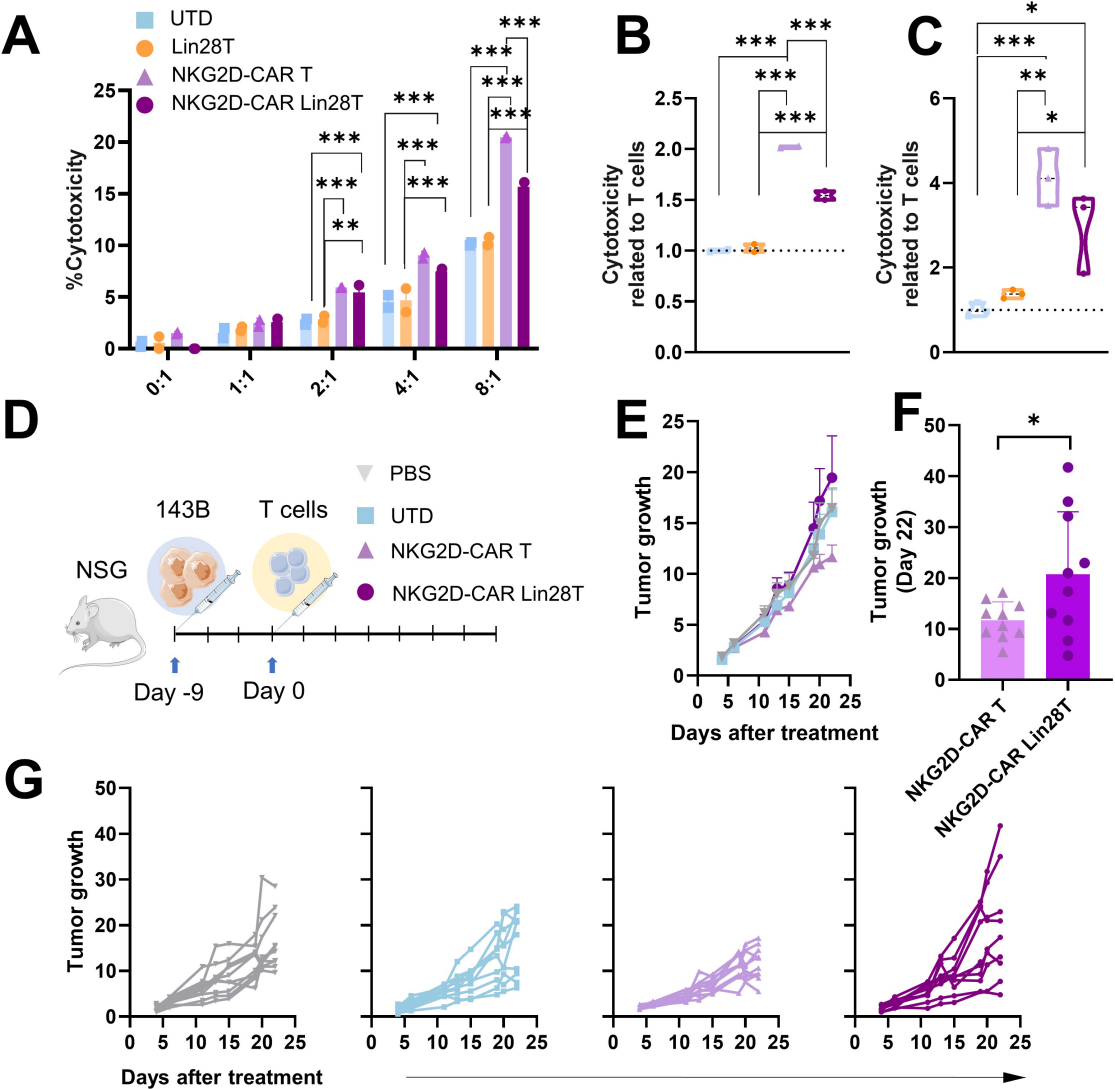
NKG2D-CAR Lin28 T cells impair NKG2D-CAR T cells cytotoxicity *in vitro* and *in vivo*. **(A)** Cytotoxicity assay *in vitro*, cells were coculture at different effector-target ratios (1:1-8:1). Cytotoxicity was measured 4h later using LDH cytotoxicity assay. **(B)** Cytotoxicity was related to T cells at 4h at 8:1 ratio in 143B and in **(C)** Mnng_hOS. The data shown represents mean ± SD. One-way ANOVA followed by Tukey’s test (n=2-3). *p<0.05, **p<0.01, ***p<0.001. **(D)** Schematic illustration of *in vivo* experimental setting. **(E)** Tumor growth of mice treated represented as mean + SEM (n=10). **(F)** Comparison of tumor growth of mice treated with NKG2D-CAR T and NKG2D-CAR Lin28T represented as mean ± SD (n=10) at day 22 post-treatment. *p<0.05 difference was found following a t-test statistical analysis. **(G)** Follow-up of tumor growth in mice treated with PBS (grey), UTD (T cells), NKG2D-CAR T (purple) and NKG2D-CAR Lin28T (dark purple) represented as individual values. Data were analyzed by One-way ANOVA followed by Tukey’s test (n=10).

To perform functional analysis, we cocultured Lin28T cells with different tumoral cell lines. First, we wanted to isolate the cytotoxicity of NKT-like cells against a cell line that lacks MHC I molecules, such as K562. Results revealed no significant differences *in vitro* between Lin28T cells and UTD (**Figure 2D**). Moreover, the cytotoxicity of human T cells expanded with different groups of cytokines showed no difference among the tested conditions. It is described that NKT cells degranulated in response to NKG2D engagement independently of their invariant TCR stimulation (18). We described no discernible variance of NKG2D between the two T subpopulations (**Figure 2E**, **Supplementary Figure 1B**). Similarly, we cocultured Lin28T against 143B cells in different effector ratios to test the cytotoxicity of CD8 T cells, resulting in similar results between conditions. Although T cells cytotoxicity was slightly increased in ratio-dependent manner, no significant differences in cytotoxicity between UTD and Lin28T were observed (**Figure 3A**). The absence of significant differences in cytotoxicity between Lin28T and UTD, regardless of the type of cytokine stimulation used, suggests that the LIN28 overexpression does not notably affect T cell cytotoxicity.

### 3.4 Upregulation of LIN28 expression impaired NKG2D-CAR T cytotoxicity activity

The impact of LIN28 upregulation on NKG2D-CAR T cells cytotoxicity both *in vitro* and *in vivo* was investigated. We did a double-transduction of T cells with NKG2D-CAR and LIN28, demonstrating a high effectivity of transduction of both transgenes (**Supplementary Figures 2B–E**). *In vitro* cytotoxicity was measured by LDH assay, with cocultures against osteosarcoma 143B and Mnng_hOS cell lines. Previously, we confirmed the expression of NKG2D ligands in the 143B and Mnng_hOS cell lines. Different E:T ratios (1:1, 2:1, 4:1 and 8:1) were tested at 4h of coculture. We observed a dose-dependent effect (**Figure 3A**). Relevantly, we observed a significant decrease of cytotoxicity in NKG2D-CAR Lin28T cells compared with NKG2D-CAR T cells (**Figures 3B, C**).

Moving to *in vivo* settings, an OS xenograft model was established, and treatments were i.v. administrated (PBS, UTD, NKG2D-CAR T, and NKG2D-CAR Lin28T) (**Figure 3D**). Subsequent monitoring of tumor growth revealed that mice treated with NKG2D-CAR Lin28T cells showed slight promotion of tumor growth compared to NKG2D-CAR T (**Figures 3E–G**). These findings collectively suggest that the upregulation of LIN28 into NKG2D-CAR T cells might compromise their cytotoxic potential both *in vitro* and *in vivo*, highlighting the critical role of LIN28 in modulating CAR T cell function and anti-tumor responses.

## 4 Discussion

LIN28 serves as a critical controller orchestrating the transition from pluripotency to differentiated cell lineages (19). According to these roles, LIN28 demonstrates proficiency in reprogramming somatic cells into pluripotent states (20). Within murine T lymphocytes, LIN28 significantly shapes early development and functionality (21). In the present study, we investigated the impact of LIN28 transduction upregulation on human T cells function, including CAR T immunotherapies.

It was published that transgenic upregulation of LIN28 in murine models leads to an aggressive peripheral T cell lymphoma (22). In humans, high LIN28B expression is associated with juvenile myelomonocytic leukemias and specific subtypes of pediatric leukemias (23, 24). However, our lentiviral transduction of LIN28 into T cells effectively induced stable expression of LIN28 without compromising cell viability or the expansion potential of human T cells *in vitro*. It is important to note that neonatal and adult T cells are derived from different progenitor cells (25). These differences in the stage of development of LIN28 overexpression might have a differential impact on their proliferative effect.

According to the literature, our findings reveal the downstream consequences of LIN28 overexpression, particularly on *let-7* miRNA family expression (26). As shown with murine T cells, LIN28 upregulation in human peripheral blood-derived T cells report a regulatory role in controlling miRNA-mediated pathways, potentially implicating LIN28 in broader regulatory networks governing T cell function and differentiation. Several lines of evidence suggest that the level of *let-7* expression appears to inversely correlate with the activation status of the T cells (27). Once T cells are exposed to antigens, T cells rapidly down-regulate *let-7* expression. This enhances the proliferation and differentiation of activated T cells into effector lymphocyte subsets (11). On the other hand, the same group has recently reported that *let-7* downregulation promotes the generation of a terminal effector population that becomes vulnerable to exhaustion and cell death in immunosuppressive environments and fails to reject tumors (12). In addition, *let-7* downregulation in fetal CD4+ T cells mediate homeostasis and self-tolerance through increased signaling through the TGF-β pathway and differentiation into Tregs (28). Considering these controversies, we wanted to investigate the role of LIN28 in T-cell-based cancer treatment.

Current cancer immunotherapies focus on strengthening the cytotoxicity function of CTLs by preventing an exhausted state of effector cells. One way to optimize manufacturing of T cells in immunotherapy is by using different types of cytokines, such as IL2, IL7, IL15, IL18 and IL21 (17). As our results, IL2, IL15 and IL21 lead the *ex vivo* expansion. Although we observed no differences between T-cell stimulation with different cytokines, other research shows differences in the differentiation status of T-cells when stimulated with different types of cytokines (29). IL2 induced a more differentiated state, whereas IL15 stimulates *ex vivo* expansion and IL21 younger phenotype (29, 30). Upon no activation differences were observed, we expanded CAR T cells with IL15 and IL21 because recent studies show that combined expression of IL-15 and IL-21 result in a less differentiated profile and longer survival in repeated exposures to tumor cells (31).

Other way to potent cytotoxicity function of T cells is via blockade of immunosuppressive ligand-receptor interactions in the tumor microenvironment (32). Some studies demonstrated that LIN28 has a potential role in tumor progression and mediated immune checkpoint upregulation, such as PD-L1 expression (33). Moreover, in this study treatment with a LIN28 inhibitor, C1632, significantly augmented the tumor-killing activity of the GPC3-CAR T cells against hepatocellular carcinoma cell (HCC) lines *in vitro* and *in vivo* through inhibition of PD-L1 and IDO in the tumor. However, our findings revealed that ectopic LIN28 expression in human T cells leads to sustained PD1 expression. Combining these results, one could speculate that the observed effect in these HCC models could also be attributed to the inhibition of LIN28 in CAR T cells and the subsequent upregulation of *let-7*, potentially enhancing their antitumoral activity.

We have analyzed the consequences of upregulated LIN28, and subsequent downregulation of *let-7* expression, within NKG2D-CAR T cells. Notably, we observed that the upregulation of LIN28 in CAR T cells attenuates their cytotoxic capabilities. Contrary to what we first hypothesized; our findings coincide with the growing interest in using miRNA *let-7* stimulators as a therapeutic tool (34). Thus, we speculate now that upregulation of *let-7* would potentially enhance the anti-tumor activity of CAR T cells. Importantly, this strategy differs from other methodologies described that potentiate *let-7* into tumor cells, which can result in unwanted effects on adjacent non-target cells, such as tumor-associated macrophages. Indeed, overexpression of *let-7c* has been associated with a shift towards an M2 phenotype associated with poor immunotherapy responses (35). Selectively potentiating *let-7* within CAR T cells might mitigate the side effects in broader systemic applications and potent its cytotoxicity. Overall, this strategy may diminish early exhaustion and improve persistent memory of CAR T.

In conclusion, our work provides a comprehensive insight into the complex interplay between LIN28 and T cell functionality. Our initial hypothesis was that Lin28 overexpression could enhance the functionality of CAR-T cells; however, our findings highlighted its potential impairment in CAR T cell functionality. Future research should continue to understand the role of upregulation *let-7* within CAR T cells, potentially opening avenues for improving the efficacy of CAR T cell therapies.

## Data Availability

The original contributions presented in the study are included in the article/**Supplementary Material**. Further inquiries can be directed to the corresponding author.

## Supplementary material

Supplementary Figure 1 LIN28 upregulation conserves T cell phenotype. **(A)** Mean fluorescence intensity (MFI) of PD1 expression on T cells. **(B)** MFI of NKG2D in CD4+ and CD8+ T cells. All data are shown as the mean ± SD. All results are representative of at least 2 independent experiments from different healthy donors. UTD: untraduced T cells (blue); Lin28T: transduced T cells (orange).

Supplementary Figure 2 Efficacy of double-transduction to generate NKG2D-CAR T cells and upregulate Lin28T. **(A)** Schematic representation for the CAR construct used. **(B)** Histogram of NKG2D expression on T cells from PBMCs. **(C)** Percentage of NKG2D expressing T cells. **(D)** Histogram of GFP expression on T cells from PBMCs. **(E)** Percentage of GFP expressing T cells. The data shown are representative of three independent experiments, mean ± SD. One-way ANOVA followed by Tukey’s test. **p<0.01, ***p<0.001. **(F)** Relative mRNA abundance of the Lin28 gene was quantified by RT-qPCR in CAR T cells at day 10 post-transduction, analyzed with t-test, **p<0.01 **(G)** LIN28 and actin expression analyzed by Western blot analysis at 10 days post-transduction. **(H)** Relative miRNA abundance of the let-7 family was quantified by steep-loop RT-qPCR in CAR T cells, analyzed with t-test, **p<0.01. UTD: untraduced T cells (blue); NKG2D-CAR T (light purple); NKG2D-CAR Lin28T (dark purple).

Supplementary Table 1 Stem-loop RT primers and qPCR primers.

Supplementary Table 2 Flow cytometer antibodies.
