# Supplementary figures and images for: LIN28 upregulation in primary human T cells impaired CAR T antitumoral activity
Source: Front Immunol. 2024 Oct 16;15:1462796. doi: 10.3389/fimmu.2024.1462796 (PMC11521810; doi:10.3389/fimmu.2024.1462796)

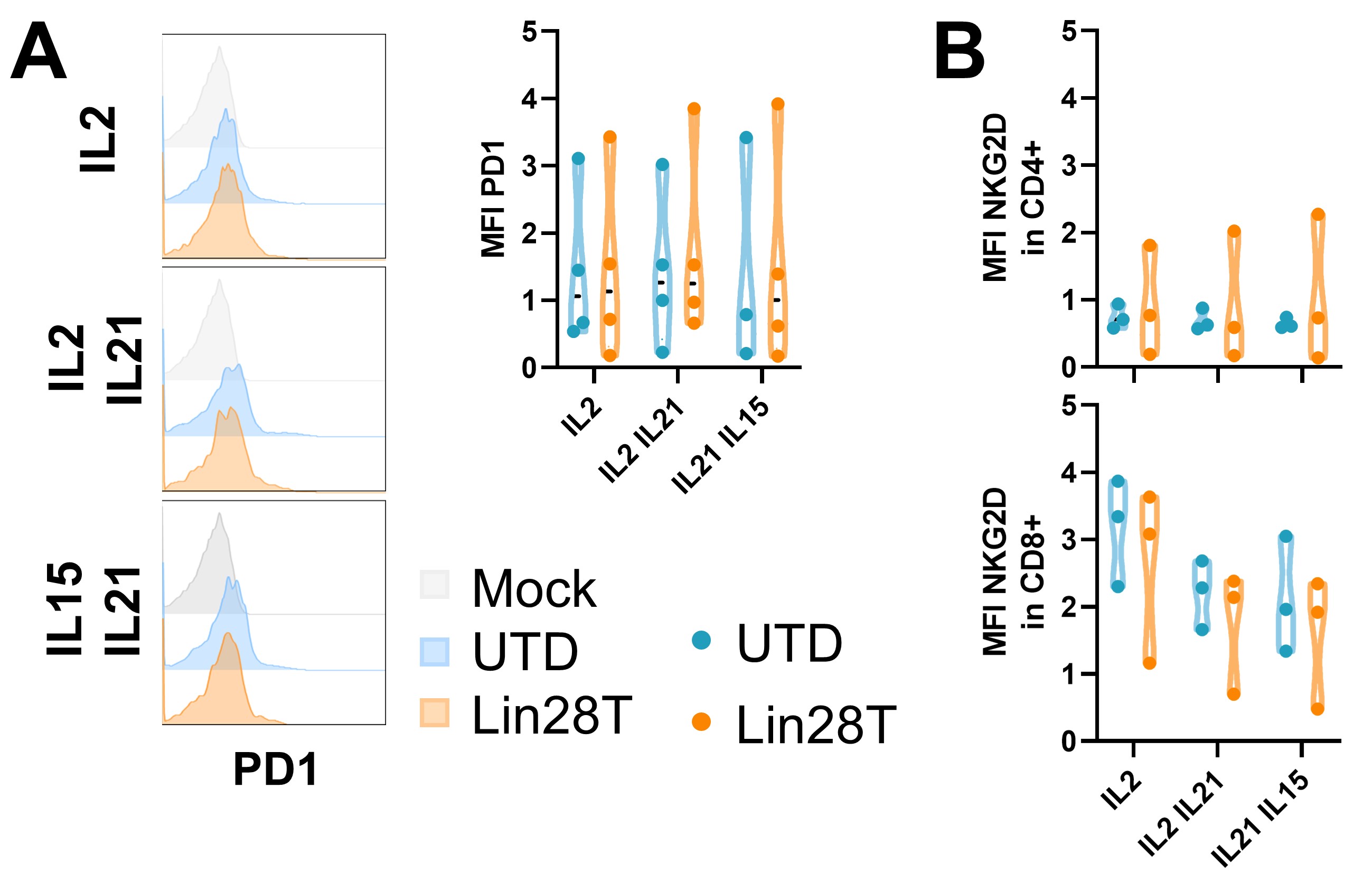

Supplement: Supplementary Figure 1 — LIN28 upregulation conserves T cell phenotype. (A) Mean fluorescence intensity (MFI) of PD1 expression on T cells. (B) MFI of NKG2D in CD4+ and CD8+ T cells. All data are shown as the mean ± SD. All results are representative of at least 2 independent experiments from different healthy donors. UTD: untraduced T cells (blue); Lin28T: transduced T cells (orange). [file Image1.jpeg]

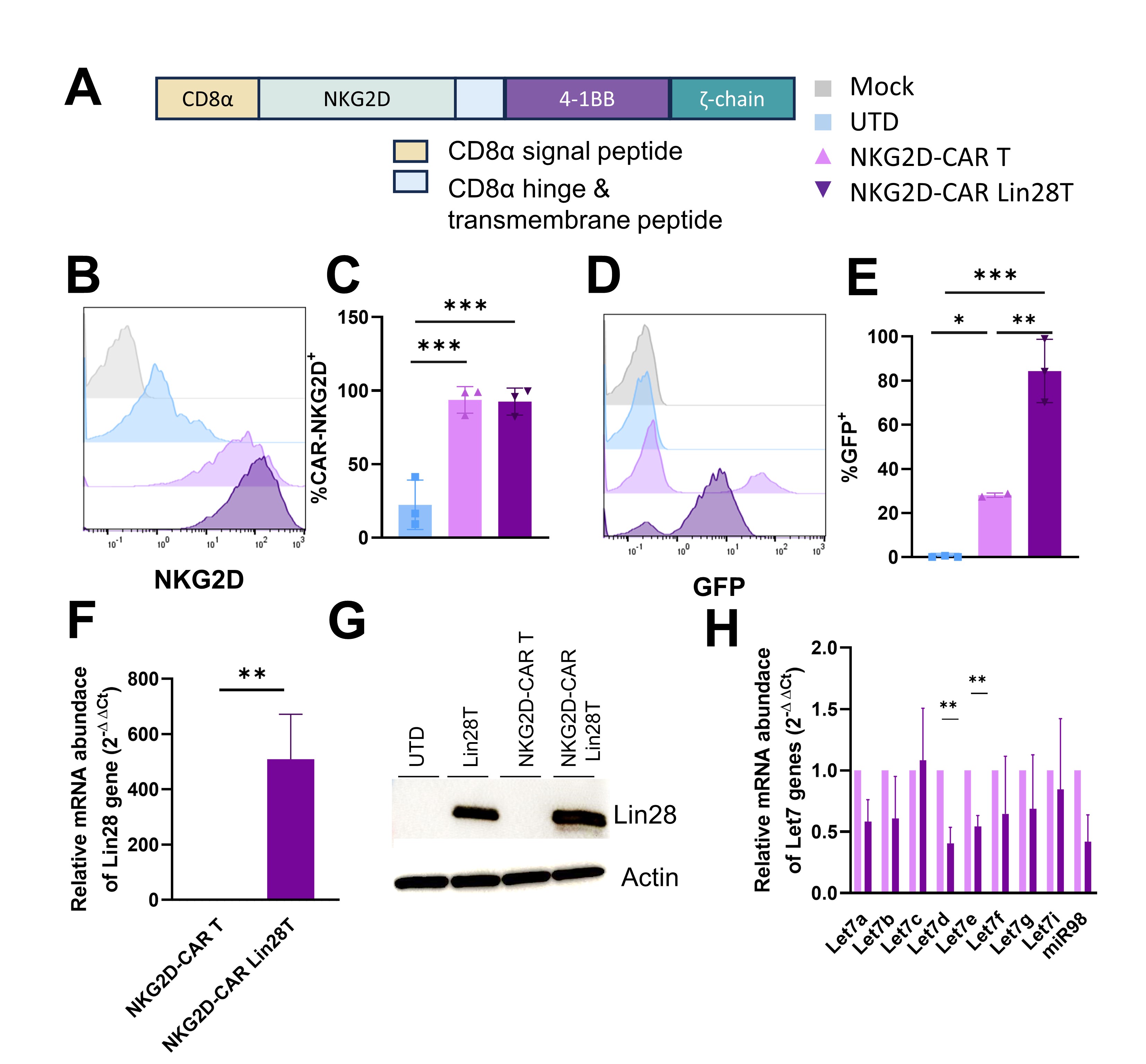

Supplement: Supplementary Figure 2 — Efficacy of double-transduction to generate NKG2D-CAR T cells and upregulate Lin28T. (A) Schematic representation for the CAR construct used. (B) Histogram of NKG2D expression on T cells from PBMCs. (C) Percentage of NKG2D expressing T cells. (D) Histogram of GFP expression on T cells from PBMCs. (E) Percentage of GFP expressing T cells. The data shown are representative of three independent experiments, mean ± SD. One-way ANOVA followed by Tukey’s test. **p<0.01, ***p<0.001. (F) Relative mRNA abundance of the Lin28 gene was quantified by RT-qPCR in CAR T cells at day 10 post-transduction, analyzed with t-test, **p<0.01 (G) LIN28 and actin expression analyzed by Western blot analysis at 10 days post-transduction. (H) Relative miRNA abundance of the let-7 family was quantified by steep-loop RT-qPCR in CAR T cells, analyzed with t-test, **p<0.01. UTD: untraduced T cells (blue); NKG2D-CAR T (light purple); NKG2D-CAR Lin28T (dark purple). [file Image2.jpeg]
