# Supplementary material for: LIN28 upregulation in primary human T cells impaired CAR T antitumoral activity
Source: Front Immunol. 2024 Oct 16;15:1462796. doi: 10.3389/fimmu.2024.1462796 (PMC11521810; doi:10.3389/fimmu.2024.1462796)
Supplement: Supplementary Table 1 — Stem-loop RT primers and qPCR primers. [file Table1.docx]

| Gene | Sequence (5’ $\boldsymbol{\to}$ 3’) |
| --- | --- |
| Stem-loop primers |  |
| U6 (RT) | CTCAACTGGTGTCGTGGAGTCGGCAATTCAGTTGAGAAAAATATG |
| Let‑7a/e/f‑5p (RT) | CTCAACTGGTGTCGTGGAGTCGGCAATTCAGTTGAGAACTATAC |
| Let‑7b‑5p (RT) | CTCAACTGGAGCTAGTTTCGTCGTAGGGCAGTTGAGAACCACAC |
| Let‑7c (RT) | GTCGTATCCAGTGCAGGGTCCGAGGTATTCGCACTGGATACGACAACCAT |
| Let‑7d‑5p (RT) | CTCAACTGGTGTCGTGGAGTCGGCAATTCAGTTGAGAACTATGC |
| Let‑7g‑5p (RT) | CTCAACTGGTGTCGTGGAGTCGGCAATTCAGTTGAGAACTGTAC |
| Let‑7i‑5p (RT) | GTCGTATCCAGTGCAGGGTCCGAGGTATTCGCACTGGATACGACAACAGC |
| miR‑98 (RT) | CTCAACTGGTGTCGTGGAGTCGGCAATTCAGTTGAGAACAATAC |
| qPCR primers |  |
| Lin28 (F) | AAGCGCAGATCAAAAGGAGA |
| Lin28 (R) | CTGATGCTCTGGCAGAAGTG |
| Let‑7a/b/c‑5p (F) | CCAGCTGGGTGAGGTAGTAGGTTGT |
| Let‑7d‑5p (F) | CCAGCTGGGAGAGGTAGTAGGTTGC |
| Let‑7e‑5p (F) | CCAGCTGGGTGAGGTAGGAGGTTGT |
| Let‑7f‑5p (F) | CCAGCTGGGTGAGGTAGTAGATTGT |
| Let‑7g/i‑5p (F) | CCAGCTGGGTGAGGTAGTAGTTTGT |
| miR‑98‑5p (F) | CCAGCTGGGTGAGGTAGTAAGTTGT |
| let‑7c/i (R) | TCCAGTGCAGGGTCCGAGGTA |
| let-7b (R) | CTGGAGCTAGTTTCGTCGTAGGG |
| Common (R3) | CTGGTGTCGTGGAGTCGGCAATT |
| GAPDH (F) | AACAGCGACACCCATCCTC |
| GAPDH (R) | CATACCAGGAAATGAGCTTGACAA |
| U6 (F) | CTCGCTTCGGCAGCACA |
| U6 (R) | AACGCTTCACGAATTTGCGT |
| RT, Reverse transcription; F, Forward; R, Reverse; Common; R3, was used as the reverse primer for the detection of let-7a/d/e/f/g. | |
