# Supplementary material for: LIN28 upregulation in primary human T cells impaired CAR T antitumoral activity
Source: Front Immunol. 2024 Oct 16;15:1462796. doi: 10.3389/fimmu.2024.1462796 (PMC11521810; doi:10.3389/fimmu.2024.1462796)
Supplement: Supplementary Table 2 — Flow cytometer antibodies. [file Table2.docx]

| **Antibody** | **Fluorochrome** | **Clone** | **Commercial** | **Reactivity** |
| --- | --- | --- | --- | --- |
| CD3 | Pacific Blue | SK7 | Biolegend | Human |
| CD3 | APC Vio770 | REA613 | Miltenyi | Human |
| CD4 | PerCP/Cyanine5.5 | OKT4 | Biolegend | Human |
| CD8 | PE | SK1 | Biolegend | Human |
| CD8 | APC Vio770 | REA734 | Miltenyi | Human |
| TCR γ/δ | APC | REA591 | Miltenyi | Human |
| CD314(NKG2D) | PE-Vio770 | REA797 | Miltenyi | Human |
| CD56 | APC | REA196 | Miltenyi | Human |
| CD279 (PD1) | PE-Vio770 | REA1165 | Miltenyi | Human |
| 7AAD viability staining solution |  |  | Biolegend |  |
